# Hyperactive Follicular Helper T Cells Contribute to Dysregulated Humoral Immunity in Patients With Liver Cirrhosis

**DOI:** 10.3389/fimmu.2019.01915

**Published:** 2019-08-13

**Authors:** Juanjuan Zhao, Jijing Shi, Mengmeng Qu, Xin Zhao, Hongbo Wang, Man Huang, Zhenwen Liu, Zhiwei Li, Qing He, Shuye Zhang, Zheng Zhang

**Affiliations:** ^1^The Second Affiliated Hospital, Southern University of Science and Technology, Shenzhen, China; ^2^Institute for Hepatology, Shenzhen Third People's Hospital, Shenzhen, China; ^3^Research Center for Clinical & Translational Medicine, Fifth Medical Center for General Hospital of PLA, Beijing, China; ^4^The Central Laboratory, The First People's Hospital of Zhengzhou, Zhengzhou, China; ^5^Department of Surgery, Fifth Medical Center for General Hospital of PLA, Beijing, China; ^6^Department for Liver Transplantation, Fifth Medical Center for General Hospital of PLA, Beijing, China; ^7^Department for Liver Transplantation, Shenzhen Third People's Hospital, Shenzhen, China; ^8^Shanghai Public Health Clinical Center and Institute of Biomedical Sciences, Fudan University, Shanghai, China; ^9^Guangdong Key Laboratory of Emerging Infectious Diseases, Shenzhen Third People's Hospital, Shenzhen, China; ^10^Key Laboratory of Immunology, School of Basic Medical Sciences, Sino-French Hoffmann Institute, Guangzhou Medical University, Guangzhou, China; ^11^Guangdong Provincial Key Laboratory of Allergy and Clinical Immunology, The Second Affiliated Hospital, Guangzhou Medical University, Guangzhou, China

**Keywords:** liver cirrhosis, cirrhosis associated-immune dysfunction, Tfh cell, humoral immunity, spleen

## Abstract

**Objectives:** Liver cirrhosis (LC) is usually accompanied by cirrhosis associated immune dysfunction (CAID), including reduced naïve T cells and memory B cells. However, little is known regarding on follicular helper T (Tfh) cell compartments in cirrhotic patients, especially in the secondary lymphoid organs such as spleen. This study characterizes splenic Tfh cells and explores its association with humoral immunity and disease progression in cirrhotic patients.

**Methods:** Using flow cytometry and histological staining, we analyzed the frequency and cytokine production of splenic Tfh cells from LC patients and healthy controls (HCs). Co-culture experiments of sorted Tfh and B cells were performed for functional analysis *in vitro*. The correlations between Tfh cells and disease progression markers as well as B cell subset perturbations were also examined.

**Results:** PD-1^high^ICOS^+^CXCR5^+^ Tfh cells were preferentially enriched in the spleen of cirrhotic patients, where they expressed higher levels of CXCR3 and produced more interleukin (IL)-21. Histologically, more splenic Tfh cells occupied the B cell follicular structure in LC patients where they shaped more active germinal centers (GCs) than those in HC spleens. *In vitro*, splenic Tfh cells in cirrhotic patients robustly induce plasma cell differentiation through IL-21 dependent manner. Finally, increased Tfh cell frequency is positively correlated with the plasma cells and disease severity in LC patients.

**Conclusions:** We conclude that hyperactive Tfh cells contribute to dysregulated humoral immunity in patients with liver cirrhosis.

## Introduction

Liver cirrhosis (LC) is the common outcome of liver inflammation induced by various etiologies such as hepatitis B virus (HBV), hepatitis C virus (HCV), alcohol, drug, and fat (1). While LC impairs the liver physiological function, it also causes cirrhosis associated immune dysfunction (CAID). CAID is a complicated process accompanied by the increased systemic inflammation and immune paralysis and/or immunodeficiency (2). The mechanisms leading to CAID remains unclear but possibly associates with the increased bacterial translocation due to the impaired gut barrier, reduced gut motility, and altered gut flora (3).

The occurrence of CAID is associated with systemic inflammation during LC disease progression, which is reflected by the persistent activation of peripheral immune cells and the elevated serum pro-inflammatory cytokines (2, 4). Compromised immune cell function was frequently reported in LC. For example, neutrophils from LC patients show impaired phagocytosis of opsonized bacteria (5). Peripheral non-classical CD14^+^CD16^+^ monocytes are increased in LC patients and display abnormal functions (6). LC profoundly depletes the circulating CD27^+^ memory B cells (7, 8). Accompanying this, hyper-globulinemia and elevated IgG and IgA levels were observed in advancing cirrhosis, irrespective of the underlying etiology (9). LC also causes T cell lymphopenia and disrupts T cell compartments. Particularly, naïve T cell depletion is more pronounced than the memory T compartment (10). Circulating natural killer (NK) cells are also compromised in liver cirrhosis and display poor responses to cytokine stimulation *in vitro* (11). These data partially define the characteristics of the CAID in peripheral blood, but little is known about its impact on the secondary lymph organ (SLO) such as spleen of cirrhotic patients, especially for T cells and B cell compartments, the two most important arms in the adaptive immune system.

Spleen is the largest SLO in the body. Spleen sits next to the liver and its blood was delivered to liver. It is well-known to play roles in immune defense against blood borne infections (12). However, spleen blood flow was congested due to portal hypertension during liver cirrhosis, resulting in splenomegaly and peripheral cytopenia (13). So far, it is unclear whether the cellular composition and underlying structure of spleen have been affected by cirrhosis. It is also debated whether the spleen plays a detrimental role in the liver pathophysiology during LC (14). Follicular T helper (Tfh) cells are one of main cell compartments of the spleen, and are usually defined as PD-1^high^ICOS^+^ CD4^+^ T cells with the expression of C-X-C motif chemokine receptor 5 (CXCR5) (15). Splenic Tfh cells play key roles in T-dependent antibody responses (16). The interaction between Tfh cells and cognate B cells induces robust B cell proliferation and instructs them to undergo differentiation through CD40 engagement and IL-21 supply (17). The persistent activation of Tfh cells is required to promote broadly and affinity matured neutralizing antibodies throughout chronic viral infection, and to adapt specificity to emerging viral variants (18). However, the high levels of Tfh cells present in chronic viral infections can also render the activation of germinal center (GC) B cells and the selection process less stringent, thus resulting in aberrant B cell activation, the generation of non-virus-specific antibodies and even autoimmune reactive antibodies, hyper-gammaglobulinemia in some cases (18). For example, in human immunodeficiency virus-1 (HIV-1) and simian immunodeficiency virus (SIV) infection, the expansion of Tfh cells present in lymph node of infected subjects correlates with hyper-gammaglobulinemia, polyclonal B cell activation, and the deletion of peripheral memory B cells (19). Similarly, the proportion of peripheral Tfh cells correlates with the emergence of autoantibodies in persistent HBV infection (20). Considering the contribution of Tfh cells to dysregulated B cell responses, the persistent interaction of Tfh cells and GC B cells is one of the key reasons for the emergence of autoreactive antibodies during autoimmune diseases (21).

Here, we hypothesized that the dysregulated Tfh cell responses might contribute to disruption of B cell compartments in cirrhosis. Our findings support the notion that the enhanced Tfh cell responses results in the persistent activation of humoral immunity, potentially depleting memory B cell pools in cirrhotic patients, and therefore is associated with LC severity.

## Materials and Methods

### Study Subjects

A total of 28 HBV associated LC (HBV-LC) patients and 23 non-HBV associated LC (non-HBV-LC) patients were recruited for this study in Shenzhen 3rd People's Hospital and in Beijing 302 Hospital. According to our described criteria previously, all patients were diagnosed (22, 23) and had not received immunosuppressive drugs within 6 months before taking samples. Forty-two age- and gender-matched individuals were enrolled as healthy controls (HCs). The study protocol was approved by the ethics committee of our institutions, and written informed consent was obtained from each subject. The basic clinical information of the enrolled individuals is listed in Table 1. Peripheral blood mononuclear cells (PBMCs) were isolated from all enrolled individuals. Spleen samples were collected from 28 HBV-LC and 13 non-HBV-LC patients with portal hypertension who underwent splenectomy. Twenty-two healthy spleen tissues were obtained from donors whose livers were used for transplantation.

**Table 1:** Basic information of enrolled subjects.

	**HC**	**HBV-LC**	**Non-HBV-LC**
Cases	42	28	23
Spleen usage^*^	22	28	13
Age (years)	48.5 (23-39)	40.3 (18-61)	46 (23-62)
Gender (M/F)	31/11	22/6	15/8
Platelet (× 10^12^)	ND	45.0 (15-97)	49.8 (28-83)
ALT (IU/L)	21.8 (12-60)	19.3 (9-50)	1.9 (10-31)
AST (IU/L)	44.1 (23-64)	25.4 (14-43)	29.6 (15-56)
Total bilirubin (μmol/L)	16.4 (10.6–29.1)	17.3 (7.3–37.9)	15.7 (3.7–29.8)
Albumin (g/L)	29.1 (22–34.5)	38.0 (32-48)	37.9 (35–42)
Prothrombin time (second)	13.9 (12.1–16.8)	13.0 (11.5–14.7)	12.9 (11.1–16.8)
Prothrombin activity	ND	68.2 (54.6–53.2)	70.2 (47.6–87.8)
HBeAg (±)	NA	6/22	NA
Child score (5/6/7)	NA	8/15/5	4/13/6

*M, male; F, female; HBeAg, hepatitis B e antigen; ND, not determined; NA, not applicable*.

* *Clinical data in HC subjects were from only donors with spleen usage*.

### Fluorescence Antibodies and Flow Cytometry

All of the fluorescence-conjugated antibodies were purchased from BD Bioscience or Pharmingen (San Diego, CA), eBioscience (San Diego, CA), and BioLegend (San Diego, CA). For T cell subset staining, allophycocyanin-Cy7 (APC-Cy7)-conjugated anti-CD45, amCyan-conjugated anti-CD3, pacific blue (PB)-conjugated anti-CD4, fluorescein isothiocyanate (FITC)-conjugated anti-CXCR5 (CD185), phycoerythrin (PE)-conjugated anti-CCR6 (CD196), peridinin chlorophyll protein-Cy5.5 (PerCP-Cy5.5)-conjugated anti-CXCR3 (CD183), phycoerythrin-Cy7 (PE-Cy7)-conjugated anti-PD-1, APC-conjugated anti-ICOS were used. For intracellular cytokine staining (ICCS), PE-conjugated anti-IL-21, APC-conjugated anti-IL-17a, and PE-Cy7-conjugated anti-IFN-γ were additionally used. For B cell subset staining, FITC-conjugated anti-CD19, PE-conjugated anti-IgD, PE-Cy7-conjugated anti-CD38, and APC-Cy7-conjugated anti-CD27 were used. The phenotypic analysis were performed at the optimal monoclonal antibody (mAb) concentrations according to the previously reported standard protocols (24). For surface marker staining, PBMCs, or spleen cells were incubated with fluorescence-conjugated surface antibodies. For intracellular staining, the cells were stained surface markers first, permeabilized and were then stained with the corresponding intracellular antibodies. Aliquots of cells were used for the analysis using FACSCanto II and FlowJo software (Tristar, San Carlos, CA). At least 50, 000 events were analyzed for per sample.

### Definition of T Cell Subsets and Analysis of Tfh Cells

According to previous studies (15), CD4^+^ and CD8^+^ T cell subsets were defined by the expression of chemokine receptors CXCR5, CCR6, and CXCR3. Among CD4^+^ T cells, CXCR5^+^ cells were defined Tfh-like cells. Within CXCR5^−^CD4^+^ T cells, CCR6^+^CXCR3^−^ cells, CCR6^+^CXCR3^+^ cells, CCR6^−^CXCR3^+^ cells, CCR6^−^CXCR3^−^ cells were defined as Th17 cells, Th1/Th17 cells, Th1 cells, and Th2 cells, respectively. Similarly, among CD4^−^ T cells (most are CD8^+^ T cells), CXCR5^+^ cells were defined Tfc-like cells, and CCR6^+^CXCR3^−^, CCR6^+^CXCR3^+^, CCR6^−^CXCR3^+^, CCR6^−^CXCR3^−^ CXCR5^−^ T cells were defined as Tc17 cells, Tc1/Tc17 cells, Tc1 cells, and Tc2 cells, respectively. Quantitation of peripheral blood Th and Tc cell subset percentages was performed in 24 HBV-LC patients, 23 non-HBV-LC patients, and 42 HCs. Among the available spleens, the splenic cells were further comprehensively analyzed for the expression of PD-1 and ICOS among the CXCR5^+^CD4^+^ T cells which were defined Tfh cells.

### Cell Stimulation

To evaluate the IFN-γ, IL-17a, and IL-21 secreting function of CD4^+^CXCR5^+^PD-1^high^ Tfh cells, spleen cells (1 × 10^6^) were cultured in a medium alone or with PMA (50 ng/ml) and ionomycin (1 μg/ml) in 96-well plate for 6 h. After 6 h incubation, the cells were collected and stained with surface and intracellular antibodies with anti-IFN-γ, anti-IL-17a, or anti-IL-21. Then the percentages of IFN-γ-, IL-17a-, or IL-21-producing cells were analyzed between HC subjects and HBV-LC patients.

### Cell Sorting

Peripheral blood mononuclear cells (1 × 10^7^) were first used for CD4^+^ T cell enrichment using microbeads (Cat# 130-096-533, Miltenyi Biotec, Germany). Then the isolated CD4^+^ T cells are stained with optimal concentrations of AmCyan-conjugated anti-CD3, PerCP-conjugated anti-CD4, FITC-conjugated anti-CXCR5, and PE-conjugated anti-PD-1 antibodies for 25 min. Alternatively, PBMCs were also stained with APC-conjugated anti-CD19. A FACS Aria II cell sorter was used to purify CD4^+^CXCR5^+^PD-1^high^ cells, CD4^+^CXCR5^+^PD-1^+^ cells, CD4^+^CXCR5^+^PD-1^−^ cells, and CD4^+^CXCR5^−^ cells as well as CD19^+^ B cells. The purity of the sorted cell subsets was more than 90%.

### The Co-culture of Tfh Cells With B Cells

Similar to previous studies (17), the sorted CD4^+^CXCR5^+^PD-1^high^ cells, CD4^+^CXCR5^+^PD-1^+^ cells, CD4^+^CXCR5^+^PD-1^−^ cells, and CD4^+^CXCR5^−^ cells from 4 HBV-LC patients or 4 HC donors were co-cultured with allogeneic or autologous CD19^+^ B cells at the ratio of 1:1 in RPMI-1640 medium containing 10% fetal bovine serum (FCS) and staphylococcal enterotoxin B (SEB) (100 ng/ml). Alternatively, rIL-21R-Fc chimera (10 μg/mL; R&D Systems, Minneapolis, MN) was added when the CD4^+^CXCR5^+^PD-1^high^ cells co-cultured with B cells (24). After 7–8 day culture, the cells were collected to assess the generation of plasma cells.

### Enzyme-Linked Immunosorbent Assays (ELISA)

An ELISA quantitative kit for the detection of human IgG and IgM (Bethyl Laboratories, Montgomery, TX) was used to measure the concentrations of total IgG and IgM from culture supernatants and plasma. The ELISA kits of human fatty acid binding protein 2 (FABP2, Cat#: DY3078) and CXCL13 (Cat#: DCX130) were purchased from R&D System Inc (Minneapolis, MN, USA), and were used measure their concentration in the plasma from LC and HC subjects.

### Immunohistochemistry

Hematoxylin-eosin (HE) and immunohistochemistry staining were performed using paraffin-embedded 4 μm tissue sections. The sections were first incubated with optimal concentrations of primary mAbs, including anti-IL-21 (abcam, ab118510), anti-CD20 (clone L26), anti-CD4 (clone IF6), and anti-PD-1 (clone NAT105) (Zhongshan Goldenbridge Biotech, Beijing, China) for 1 h at RT. Then the sections were incubated with biotinylated goat-rabbit, goat anti-rat, or biotinylated rabbit anti-mouse antibodies (Zhongshan Goldenbridge Biotech, Beijing, China), respectively. The GC was observed independently by two pathologists from 5 of representative high-power fields (200 × magnification) (24).

### Correlation Analysis of Splenic Tfh Cells and B Cell Subsets

To analyze the correlation between splenic PD-1^high^ICOS^+^CXCR5^+^CD4^+^ Tfh cells and relevant B-cell subsets, 28 of HBV-LC patients with available spleen were studied. The correlations between the percentage of Tfh cells and total B cells or B cell subsets or plasma IgG and IgM levels were analyzed. CD19^+^CD10^−^CD38^lo^ mature B cells were divided into IgD^+^CD27^−^ naïve B cells (naïve B cells), IgD^+^CD27^+^ marginal zone B cells (MZBs), and IgD^−^CD27^+^ class-switched memory B cells (cMBCs) and IgD^−^CD27^−^ atypical memory B cells (aMBCs). CD19^+^IgD^−^CD38^high^ phenotype defined the plasmablasts (24).

### Correlation Analysis of Splenic Tfh Cells and Liver Functions

The correlations between the percentage of spleen Tfh cells and several clinical biochemical parameters including serum albumin (ALB), total bilirubin (TBIL), and prothrombin activity (PTA) and Child-Pugh scores were analyzed in the 28 HBV-LC patients. In addition, we also evaluated the levels splenic Tfh percentages in HBV-LC patients with various Child-Pugh scores.

### Statistical Analysis

All statistics were analyzed using SPSS 16.0 software. The data are presented as mean values and standard deviations. Multiple comparisons were first performed among the different groups using the non-parametric Kruskal–Wallis *H*-test. Comparisons between various groups were made using the Mann–Whitney *U*-test, whereas comparisons between the same individual were made using the Wilcoxon's matched-pairs test. The correlations between two variables were analyzed using the Spearman rank correlation test. *P* < 0.05 at two-sides was considered to be significant for all analyses.

## Results

### CXCR5^+^ CD4 Tfh-like Cells Are Enriched in Spleen and Peripheral Blood in LC Patients

**Figure 1:**
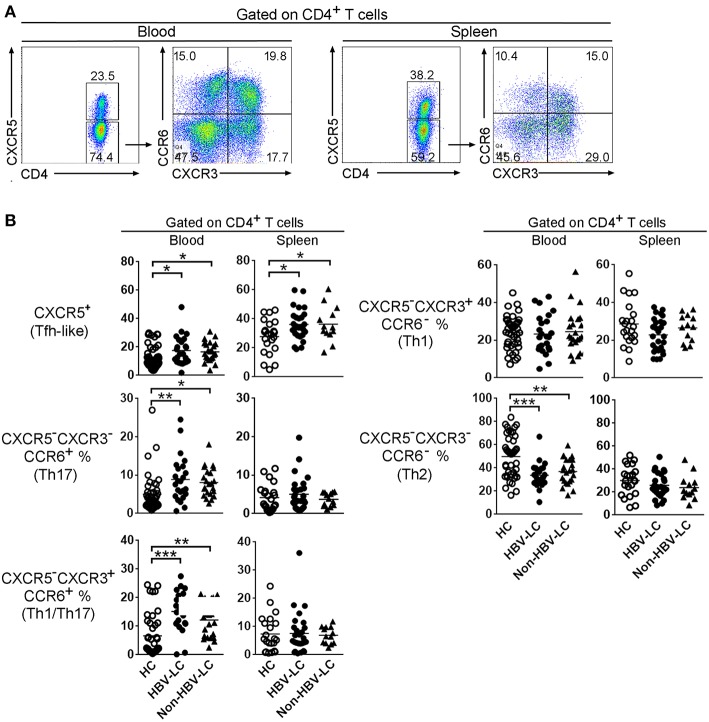
Disruption of Th cell subset distribution in patients with liver cirrhosis. **(A)** Representative dot plots show the gating strategy of different CD4^+^ subsets basing on CXCR5, CCR6, and CXCR3 expression in spleen and peripheral blood. Values in the quadrants represent the percentages of each subset. Among CD4^+^ T cells, CXCR5^+^ marks Tfh-like cells. For CXCR5^−^CD4^+^ T cells, CCR6^+^CXCR3^−^ Th17 cells, CCR6^+^CXCR3^+^ Th1/Th17cells, CCR6^−^CXCR3^+^ Th1 cells, CCR6^−^CXCR3^−^ Th2 cells were defined, respectively. **(B)** Summarized data show the percentages of CD4^+^ T cell subsets in the peripheral blood from HC (*n* = 42), HBV-LC (*n* = 24), and Non-HBV-LC groups (*n* = 23) and in the spleen from HC (*n* = 22), HBV-LC (*n* = 27), and Non-HBV-LC groups (*n* = 13). Each dot represents one subject, and line represents mean value. ^*^P < 0.05, ^**^P < 0.01, ^***^P < 0.001, Mann–Whitney *U*-test.

We first analyzed the frequencies of peripheral and splenic CD4^+^ Th (Figure 1A) and CD8^+^ Tc (Supplemental Figure 1A) cell subsets using flow cytometry according to the expression of chemokine receptors CXCR5, CCR6, and CXCR3. As shown in Figure 1B, among peripheral CD4 T cells, the percentages of Tfh-like cells, Th1/Th17 cells, and Th17 cells in HBV-LC and non-HBV-LC patients were all significantly higher than those in HC subjects (*P* < 0.05). By contrast, Th2 cell percentages were significantly decreased (*P* < 0.001) and Th1 cells were comparable between LC patients and HC subjects. Similar trends were also found among peripheral CD8^+^ Tc subsets (Supplemental Figure 1B). Notably, when splenic CD4^+^ and CD8^+^ T cell subsets were analyzed, it is found that only Tfh-like cell proportions were significantly increased in LC patients than those in HC subjects; while other CD4^+^ and CD8^+^ T cell subsets were unchanged (Figure 1B and Supplemental Figure 1B). These data indicate that CXCR5^+^CD4^+^ Tfh-like cells are preferentially enriched in both peripheral blood and spleen from HBV-LC and non-HBV-LC patients.

### Tfh Cells Are Significantly Expanded With High Levels of CXCR3 Expression in the Spleen of LC Patients

**Figure 2:**
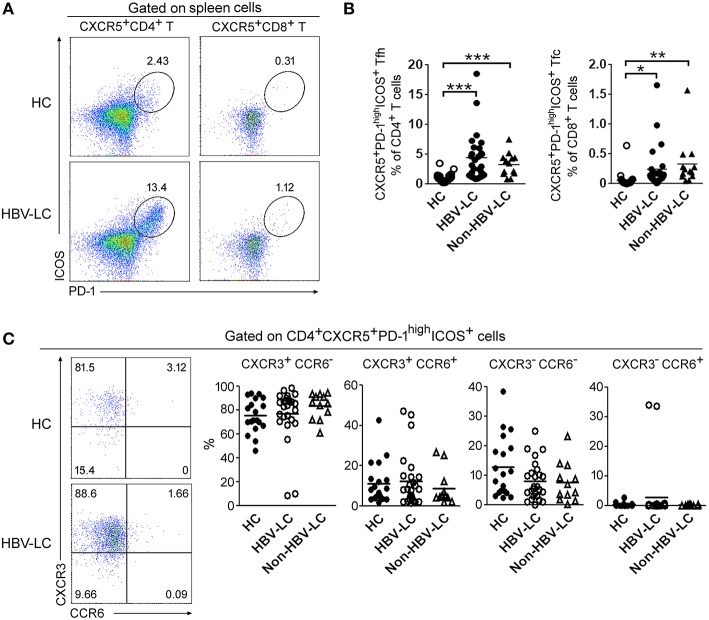
Splenic Tfh cells were preferentially expanded in LC patients. **(A)** Representative dot plots show the proportion of PD-1^high^ICOS^+^ cells among CXCR5^+^CD4^+^ Tfh-like cells and CXCR5^+^CD8^+^ Tfc-like cells in spleen from a LC patient. Values in the quadrants represent the percentages of splenic Tfh and Tfc cell subset. **(B)** Summarized data show the percentages of PD-1^high^ICOS^+^ CXCR5^+^CD4^+^ T cells and PD-1^high^ICOS^+^ CXCR5^+^CD8^+^ T cells in the spleen from HC (*n* = 22), HBV-LC (*n* = 27), and Non-HBV-LC groups (*n* = 13). **(C)** CCR6 and CXCR3 expression within splenic PD-1^high^ICOS^+^CD185^+^CD4^+^ Tfh cells. Representative dot plots show the expression of CCR6 and CXCR3 when gated on splenic PD-1^high^ICOS^+^CXCR5^+^CD4^+^ cells in spleen from a LC patient (Left). Values in the quadrants represent the percentages of each subset. Summarized data (Right) show the percentages of Tfh cell subsets in the spleen from HC (*n* = 22), HBV-LC (*n* = 27), and Non-HBV-LC groups (*n* = 13). **(B,C)** Each dot represents one subject, and line represents mean value. ^*^P < 0.05, ^**^P < 0.01, ^***^P < 0.001, Mann–Whitney *U*-test.

PD-1 and ICOS are recognized as main markers to define Tfh cells, we therefore analyzed their expression in these CD4^+^ Th cell and CD8^+^ Tc cell subsets from both peripheral blood and spleen (Supplemental Figure 2). PD-1^high^ICOS^+^ cells formed a distinct population within the splenic Tfh-like cells; their numbers in peripheral Tfh-like cells and splenic Tfc cells were much fewer and almost absent from other peripheral and splenic CD4^+^ and CD8^+^ T cell subsets (Supplemental Figure 2). We then compared the percentages of PD-1^high^ICOS^+^CXCR5^+^ Tfh cells in LC patients and HC subjects (Figure 2A and Supplemental Figure 3A). We found that the proportion of PD-1^high^ICOS^+^CXCR5^+^ Tfh cells was significantly increased in both spleen (Figure 2B) and peripheral blood (Supplemental Figure 3B) from LC patients compared to HC subjects (both *P* < 0.01). PD-1^high^ICOS^+^CXCR5^+^ Tfc cells were also significantly increased in the spleen of LC patients (Figure 2B) compared to those from HC subjects (*P* < 0.01) but not in blood (Supplemental Figure 3B). These data indicated that PD-1^high^ICOS^+^CXCR5^+^ Tfh cells are preferentially enriched in the splenic CD4^+^ T cells, which is further increased in LC patients.

We further analyzed the proportion of CXCR3- and CCR6-expressing cells in splenic Tfh cells from LC and HC subjects (Figure 2C). It is found that CXCR3^+^ cells and CCR6^+^ cells account for nearly 80 and 10% of splenic PD-1^high^ICOS^+^ Tfh cells, respectively, regardless of disease status (Figure 2C). The proportion of CXCR3^−^CCR6^−^ Tfh2 cells was higher among CXCR5^+^ Tfh-like cells in both spleen and blood from HC donors vs. LC patients. The CXCR3^+^CCR6^−^ Tfh1 cells was significantly expanded in the spleen of LC patients. While in peripheral blood, the proportion of CCR6^+^ cells (including both CXCR3^+^CCR6^+^Tfh1/Tfh17 cells and CXCR3^−^CCR6^+^ Tfh17 cells) were significantly increased in LC patients (Supplemental Figures 4A,B). These data indicated that splenic Tfh-like cells expressed higher levels of CXCR3 in LC patients.

### Splenic Tfh Cells From LC Patients Produced Higher Levels of IL-21

**Figure 3:**
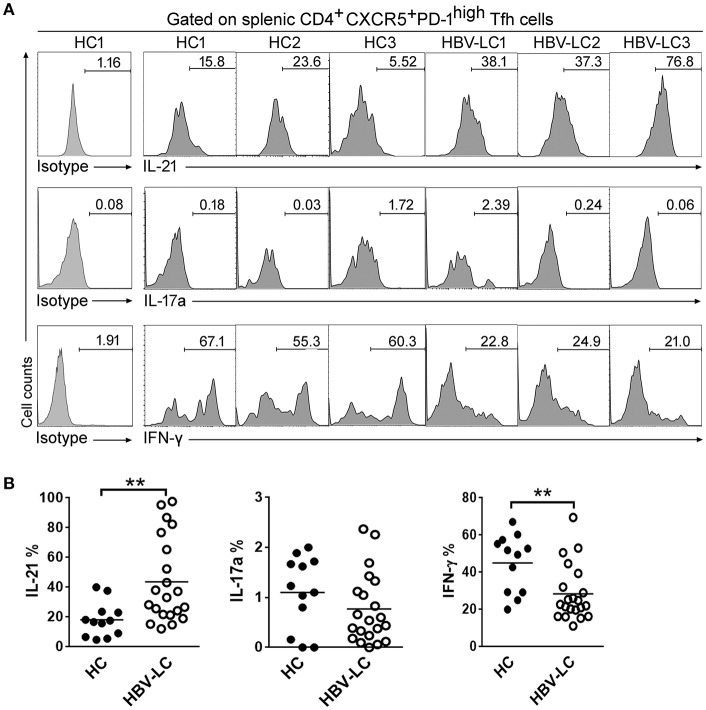
Increased IL-21 production by splenic PD-1^high^ICOS^+^CXCR5^+^CD4^+^ Tfh cells in LC patients. **(A)** Representative histograms from 3 HBV-LC and 3 HC subjects reflect IL-21, IFN-γ, and IL-17a production by splenic PD-1^hi^ICOS^+^CXCR5^+^CD4^+^ Tfh cells in responses to PMA/ionomycin. **(B)** Pool data indicated that splenic PD-1^hi^ICOS^+^CXCR5^+^CD4^+^ Tfh cells release more IL-21, less IFN-γ, and similar levels of IL-17a in HBV-LC (*n* = 18) than in HCs (*n* = 10) when stimulated with PMA/ionomycin. Each dot represents one subject, and line represents mean value. ^**^P < 0.01, Mann–Whitney *U*-test.

Next, we analyzed cytokine production by splenic Tfh cells including IL-21, IFN-γ, and IL-17a in responses to PMA/ionomycin *in vitro* (Figure 3A). As shown in Figure 3B, the levels of IL-21 produced by splenic Tfh cells in HBV-LC patients were significantly higher than those in HCs (*P* < 0.05). IFN-γ production by splenic Tfh cells was significantly reduced in HBV-LC patients as compared to HC subjects. There is no significant difference for IL-17a production by splenic Tfh cells in LC patients as compared to those in HC subjects. These data indicated the splenic Tfh cells produced more IL-21 in LC patients, which may shape the specific microenvironment promoting humoral immunity when liver disease progresses into cirrhotic phases.

### More Splenic Tfh Cells Moved Into B Cell Zone and Shaped Active Germinal Centers (GCs) in LC Patients

**Figure 4:**
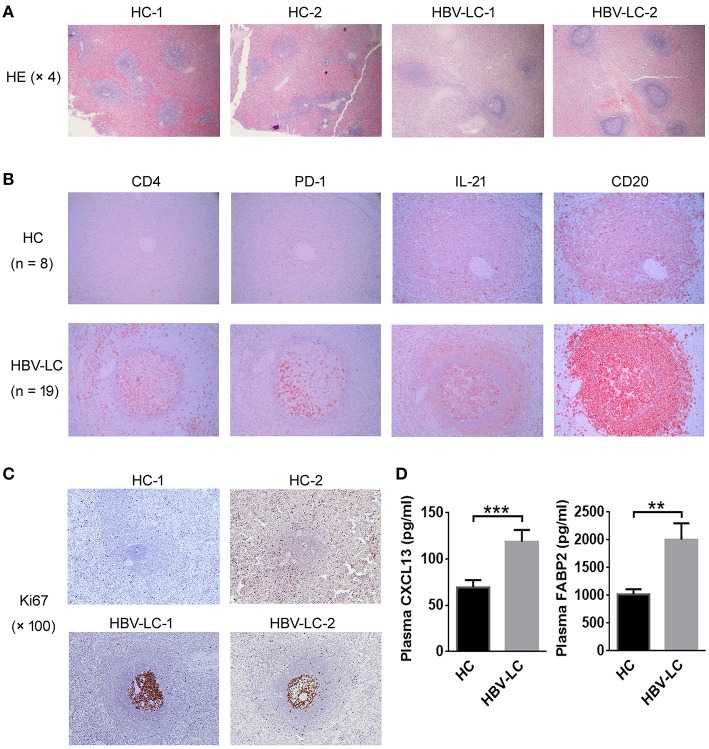
More splenic Tfh cells are migrated in the MZ region in LC patients. **(A)** Representative HE staining indicated obvious follicular-like structure in the spleen of 2 LC patients as compared to 2 HC subjects. **(B)** Immunohistochemical staining detected the expression of CD4, PD-1, IL-21, and CD20 in the continuous tissue sections from an HC subject and an HBV-LC patient (magnification, 200 ×). Obvious CD4^+^, PD-1^+^, and IL-21^+^ cells infiltrated into B cell MZ region in LC patients. Similar data were also found in more LC patients (*n* = 19) and HC donors (*n* = 8). **(C)** Representative Ki67 staining indicated obvious proliferation of GC B cells in the spleen of 2 LC patients as compared to 2 HC subjects. **(D)** Plasma CXCL13 and FABP2 concentrations in HBV-LC (*n* = 27) and HC subjects (*n* = 22). The data are shown as means and standard deviations. ^**^P < 0.01, ^***^P < 0.001, Mann–Whitney *U*-test.

We investigated the distribution of splenic Tfh cells using immunohistochemical staining. More follicle-like structure showing larger active GCs was present in the spleen of LC patients than that in HC spleen where the GCs were mostly degenerative. Outside of the GCs, a ring-like layer densely populated by smaller-sized cells (likely the reported mantle zone formed mostly by naive B cells) was present in LC spleen and absent from HC spleen (Figure 4A). We further stained several classical markers for Tfh cells and B cells in spleen including CD4, PD-1, and IL-21 as well as CD20 using continuous section so as to observe the localization of Tfh cells and B cells *in-situ* from LC patients vs. HC subjects. CD20 staining indicated that the follicles were indeed splenic B cell zones. It is found that less CD4^+^, PD-1^+^, and IL-21^+^ Tfh cells were present in healthy spleen. By contrast, more PD-1^+^ and IL-21^+^ cells were accumulated within the GC area in LC patients (Figure 4B). Consistent with the role of Tfh cells in promoting GC responses, we observed significantly increased follicular Ki67^+^ B cells in LC spleens as compared to HCs (Figure 4C). Plasma CXCL13, a B cell attracting cytokine, has been considered as a marker indicating GC activity (25). We measured the CXCL13 levels in the plasma of HBV-LC and HC subjects, and found that plasma CXCL13 levels was significantly higher in HBV-LC patients than that in HC subjects (Figure 4D). Simultaneously, the levels of plasma FABP2, a marker indicating active bacterial translocation, were also increased in HBV-LC patients than that in HC subjects (Figure 4D). These data clearly indicated that more splenic Tfh cells moved into B cell zone in the spleen and shaped more active GCs in LC patients.

### The Levels of Tfh Cells Were Positively Correlated With Increased Plasma Cells in the Spleen of HBV-LC Patients

**Figure 5:**
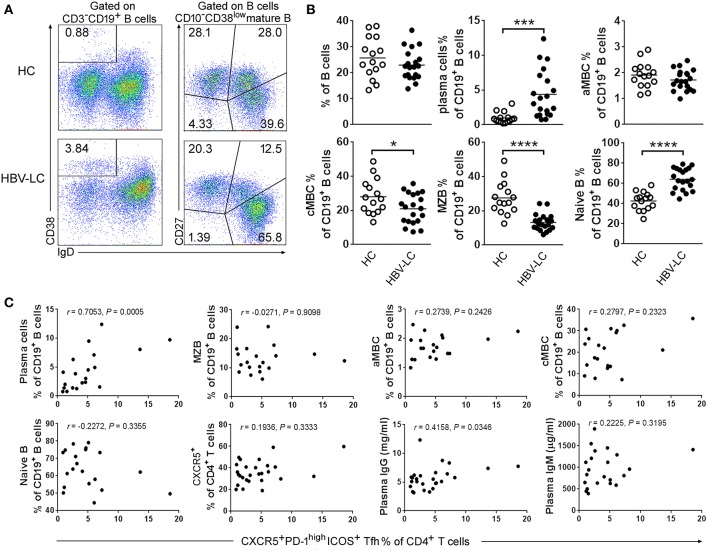
Disruption of B cell subset distribution in LC patients. **(A)** Representative dot plots show the gating strategy of B cell subsets in spleen from an LC patient. Values in the quadrants represent the percentages of each subset. Within CD3^−^CD19^+^ B cells, IgD^−^CD38^high^ plasma cells, IgD^−^CD27^+^ cMBC cells, IgD^+^CD27^+^ MZB cells, IgD^+^CD27^−^ naïve B cells, and IgD^−^CD27^−^ aMBC cells were defined, respectively. **(B)** Summarized data show the percentages of B cell subsets in the spleen from HC (*n* = 15) and HBV-LC groups (*n* = 20). Each dot represents one subject, and line represents mean value. ^*^P < 0.05, ^***^P < 0.001, ^****^P < 0.0001, Mann–Whitney *U*-test. **(C)** Correlation analysis between splenic Tfh cells and B cell subsets. The percentages of splenic Tfh cells were significantly positively with plasma cell proportion and plasma IgG levels but not with MZB, aMBC, cMBC, naïve B, splenic CXCR5^+^ CD4 T cell subsets, and plasma IgM levels in HBV-LC patients (*n* = 20). Each dot represents one subject. Spearman rank correlation test was used: *r*, correlation coefficient; *P*-values are shown.

To further explore the relationships between the increased Tfh cells and perturbated B cells, we analyzed the proportion of splenic B cell subsets in LC patients and HC subjects. CD19^+^ B cells include IgD^−^CD38^high^ plasma cells, IgD^−^CD27^+^ cMBCs, IgD^+^CD27^+^ MZBs, IgD^+^CD27^−^ naïve B cells, and IgD^−^CD27^−^ aMBCs (Figure 5A). Pooled data indicated that the percentages of plasma cells and naïve B cells are significantly increased in the spleen of LC patients as compared to those in HC subjects. In contrast, the percentages of MZBs and cMBCs were significantly decreased in the LC patients (Figure 5B), indicating that splenic B cell subsets were perturbed in LC patients.

We then investigated whether the disturbed B cell proportion was associated with the increased Tfh cells in these HBV-LC patients. It is found that the levels of splenic Tfh cells were positively associated with plasma cells (defined as IgD^−^CD38^high^CD19^+^ B cells) in LC patients (*r* = 0.7053, *P* = 0.0005, *n* = 20; Figure 5C). Although, splenic cMBC and MZB cell percentages were decreased in HBV-LC patients, there was no significant correlation between the percentages of splenic Tfh cells and the percentages of most of B cell subsets including MZBs, aMBCs, cMBCs, naïve B cells, and CXCR5^+^CD4^+^ T cells. In addition, we also found that splenic Tfh cell proportion was correlated with plasma total IgG (*r* = 0.4158, *P* = 0.0346, *n* = 20) but not IgM levels (Figure 5C). These data suggested that splenic B cell subsets were disturbed in LC patients, particularly the increased plasma cells were found to be positively associated with increased Tfh cells in the spleen of HBV-LC patients.

### Splenic Tfh Cells Promote Plasma Cell Differentiation Dependent on IL-21 in LC Patients

**Figure 6:**
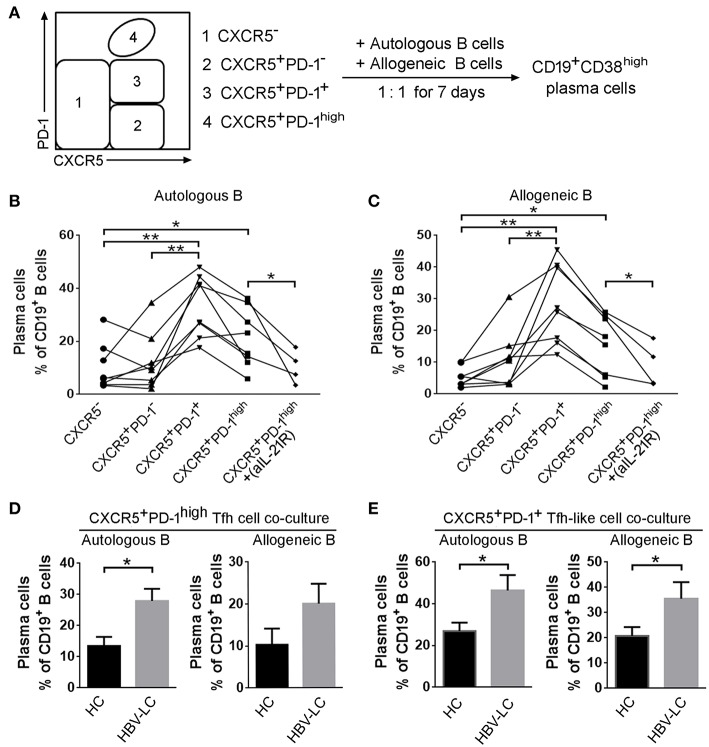
Splenic Tfh cells from HBV-LC patients have more potential to induce B cell differentiation into plasma cells *in vitro*. **(A)** Flowchart indicated that splenic CXCR5^−^CD4^+^ T cells, CXCR5^+^PD-1^+^CD4^+^ T cells, CXCR5^+^PD-1^−^CD4^+^ T cells, and CXCR5^+^PD-1^high^ Tfh cells were sorted and co-cultured with allogeneic or autologous CD19^+^ B cells at the ratio of 1:1 for 7 days, respectively. Then the percentage of C19^+^CD38^high^ plasma cells was detected. **(B,C)** Summary data indicated that the proportion of plasma cells induced by each type of CD4^+^ T cell subsets from HBV-LC patients (*n* = 5) and HC subjects (*n* = 4) in autologous **(B)** or allogeneic **(C)** CD19^+^ B cell co-culture. ^*^P < 0.05, ^**^P < 0.01, Wilcoxon's matched-pairs test. **(D,E)** Pool data showed that the proportion of plasma cells induced by Tfh cells **(D)** and CXCR5^+^CD4 Tfh-like cells **(E)** and in autologous or allogeneic CD19^+^ B cell co-culture in HBV-LC patients (*n* = 5) and HC subjects (*n* = 4). The data are shown as means and standard deviations. ^*^P < 0.05, Mann–Whitney *U*-test.

The immunological outcome of functionally increased splenic Tfh cells remains unclear in LC patients. Tfh cells moved to the B-cell follicles in LC patients where Tfh cells may interact with GC-B cells. A significantly positive correlation between splenic Tfh cells with plasma cells and serum IgG levels also indicated that splenic Tfh cells have potentials to induce naive B cells to differentiate into plasmablasts in HBV-LC patients. We therefore investigated the influence of expanded splenic Tfh cells on B cells in LC patients. Splenic CXCR5^−^CD4 T cells, CXCR5^+^PD-1^+^ CD4 T cells, CXCR5^+^PD-1^−^ CD4 T cells, and CXCR5^+^PD-1^high^ Tfh cells were co-cultured with allogeneic or autologous CD19^+^ B cells from LC or HC donors for 7 days, respectively (Figure 6A). rIL-21R-Fc fusion protein was added in CXCR5^+^PD-1^high^ Tfh cells co-culturing wells to block IL-21 signal. As shown in Figure 6B, plasma cell proportion was significantly increased in both CXCR5^+^PD-1^+^ CD4^+^ T cell and Tfh cell co-culture systems compared to CXCR5^−^CD4^+^ T cell and CXCR5^+^PD-1^−^ CD4^+^ T cell with autologous (Figure 6B) and allogeneic (Figure 6C) B cells. Blocking IL-21 signaling using rIL-21R-Fc led to the decreased proportion of plasma cells in both autologous and allogeneic B cell co-cultures *in vitro* (Figures 6B,C). Compared to HC subjects, we noted that splenic Tfh cells from LC patients induced more plasma cells differentiation in both autologous and allogeneic co-culture systems than that from healthy subjects (*P* < 0.05, Figure 6D). Similar data were also found when splenic CXCR5^+^ CD4 Tfh-like cells from LC or HC subjects were co-cultured with autologous or allogeneic B cells (Figure 6E). These data suggest that splenic Tfh cells from LC patients could promote more plasma cell differentiation dependent on IL-21.

### The Increased Splenic Tfh Cells Are Correlated With Disease Severity in HBV-LC Patients

**Figure 7:**
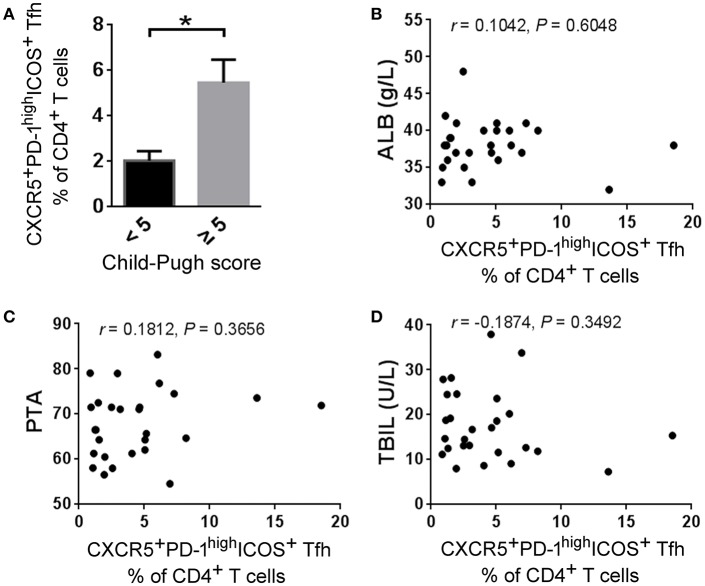
Splenic Tfh cells correlate with disease severity in HBV-LC patients. **(A)** Higher frequencies of splenic Tfh cells were present in HBV-LC patients with child score more than 5 (*n* = 19) than in those with child score <5 (*n* = 8). The data are shown as means and standard deviations. ^*^P < 0.05, Mann–Whitney *U*-test. **(B–D)** The correlation analysis between the percentages of splenic Tfh cells and clinical markers such as ALB **(B)**, PTA **(C)**, and TBIL **(D)** levels in HV-LC patients (*n* = 27). Spearman rank correlation test was used: *r*, correlation coefficient; *P*-values are shown.

We finally analyzed the correlations between splenic Tfh cell proportion and several clinical markers reflecting disease severity such as Child-Pugh score, ALB, PTA, TBIL, et al. We found that HBV-LC patients with higher Child-Pugh scores displayed more splenic Tfh cell proportion than those with lower scores (Figure 7A), although there were no significant correlations between splenic Tfh cell proportion and the levels of ALB, PTA, and TBIL (Figures 7B–D). These data indicated that expanded splenic Tfh cells are positively correlated with cirrhotic-complications in HBV-LC patients.

## Discussion

Little is known about the immune characteristics of CAID in the SLO such as spleen in cirrhotic patients, especially for Tfh and B cells acting as two important arms in the human immune system. Here, we demonstrated that splenic Tfh cells were expanded in B cell follicular region of cirrhotic patients, where they expressed higher levels CXCR3 and produced more IL-21. Thus, splenic Tfh cells induced B cells to robustly differentiate into plasma cells through IL-21, and correlated with cirrhotic severity in LC patients. This study revealed novel immune properties of CAID and highlighted the immune consequences of the interaction between Tfh and B cells in LC patients.

Our data provides novel insights to the splenic Tfh cell compartments in cirrhosis. We examined the splenic and peripheral CD4 and CD8 T cell subsets based on CXCR5, CXCR3, and CCR6 expression from LC and HC subjects. In the spleen, CXCR5^+^CD4^+^ Tfh-like cells were significantly increased in LC patients as compared to those in HC subjects. More important, PD-1^high^ICOS^+^ Tfh cells are specifically accumulated within the splenic Tfh-like cell subset rather than other Th and Tc subsets including the CXCR5^+^ CD8 T cells which play antiviral roles in SLOs (26). Although these splenic PD-1^high^ICOS^+^ Tfh cells showed substantial heterogeneity, they preferentially expressed higher levels of CXCR3. In addition, these splenic Tfh cells are largely expanded and produced more IL-21 in LC patients than those from HC subjects regardless of cirrhosis-associated etiology. This comprehensive analysis highlights splenic Tfh cells from LC patients are expanded and hyper-activated.

The immunological consequences of the increased splenic Tfh cells remained unclear in LC patients. Previous studies have indicated that the decreased T cell immunity was generally accompanied by enhanced humoral immune responses in various chronic inflammatory conditions (18). Here, our data support the notion that the expanded splenic Tfh cells may promote B cell differentiation in LC patients. We provide several evidences including the extensive co-localization and the significant positive correlation between splenic Tfh cells and plasma cell subsets as well as the potential of Tfh cells inducing B cell differentiation *in vitro*. The contribution of hyperactive Tfh cells to plasma cell differentiation may lead to the increased levels of non-specific antibodies and autoimmune antibodies during cirrhosis. It is reported that cirrhotic patients manifested hyper-immunoglobnemia (9) and had more anti-microbial antibodies than control subjects (27). Therefore, the enhanced systemic inflammation and Tfh cell immunity in cirrhotic patients may break the barrier tolerance and facilitate the induction of anti-microbial antibodies, which consequently affect the normal symbiosis of gut flora and lead to the increased microbial translocation. The reported dominant elevation of IgA type antibodies over IgG and IgM also suggested that compromised mucosal immunity could play roles in inducing anti-microbial antibodies during cirrhosis (27). Enhanced Tfh cell responses may also result in increased autoantibodies production, and HBV-infected patients with autoantibodies also showed more activated Tfh cell phenotypes than the patients without autoantibodies (20). Whether the increased non-specific and autoantibodies have detrimental impact on cirrhosis associated pathophysiology needs future studies.

Enhanced Tfh immune responses may deplete memory B cell pool through inducing more B cells differentiating into plasma cells. Recently, extra-follicular Tfh-like cells located at the border of the T cell zone and B cell follicle in human tonsils were reported to promote memory B cells to produce antibodies via CD40L and IL-21 (17). Here, we observed that Tfh cells in both follicular and extra-follicular regions in spleen from cirrhotic patients are significantly expanded and may hasten the B cell differentiation process. This finding was reminiscent with the observations in other chronic autoimmune diseases. For instance, patients with primary Sjogren's syndrome and systemic lupus erythermatosus had an expansion of peripheral Tfh-like cells with the increased IL-21 production, along with decreased CD27^+^ memory B cells and increased plasma cells, indicating prominent role of Tfh cells in aberrant distribution of B cell subsets (28, 29). These data suggest that splenic functionally enhanced Tfh cells may expedite memory B cell differentiating into plasma cells, deplete memory B cell pool, and induce non-specific antibody production.

The factors leading to functional activation of Tfh cells in cirrhotic spleen are unclear, however, it likely relates to systemic inflammatory responses in LC. Recently, the bacterial mRNA recognition is reported to increase Tfh cell differentiation, and enhances antibody responses (30, 31). The activation of the MyD88-dependent TLR8 signaling on human monocytes initializes the expression of IL-12, a main cytokine inducing Tfh cell differentiation. Human IL-1β is produced in response to bacteria and affects Tfh differentiation (32). Therefore, sensing viable bacteria by monocytes may serve as a trigger to induce Tfh cell differentiation, subsequent class-switch of B cells and antibody production. During cirrhosis, there is episodic bacterial translocation and persistent immune cell activation, as indicated by the increased FABP2 and CXCL13 levels in our study. In addition, the expansion of the Tfh cells could also be driven by IL-6 signaling derived from follicular dendritic cells and the persistent viral antigen in the host environment (33). Thus, the activation of innate immune cells including activated dendritic cells, increased inflammatory cytokines and viable microbes are all present during liver cirrhosis. We think that these combined effects may eventually promote an enhanced Tfh cell response in LC patients, especially during advanced diseases.

This study is limited by several aspects. First, we have not performed the functional and clinical analysis of splenic Tfh cells from non-HBV-LC patients although splenic Tfh cells were significantly increased in both HBV-LC patients and non-HBV-LC patients. Second, it is unclear whether the increased Tfh cells correlated with the levels of autoantibodies in LC patients, which may generate a novel understanding on CAID. Third, clinical significance has not been fully addressed in the study. Nonetheless, we reported that the hyperactive Tfh cells contribute to dysregulated humoral immunity in LC patients. Our study indicated that targeting Tfh cells may provide a way to ameliorate CAID.

## Data Availability

The datasets generated for this study are available on request to the corresponding author.

## Author Contributions

JZ, SZ, and ZZ designed the study. JZ, JS, MQ, and ZZ did the experiments and analyzed the flow cytometry data. MH provided help with IHC experiments. XZ checked case history. HW, ZLiu, QH, and ZLi provided human samples. SZ and ZZ wrote the manuscript.

### Conflict of Interest Statement

The authors declare that the research was conducted in the absence of any commercial or financial relationships that could be construed as a potential conflict of interest.

## References

[1] Tsochatzis EA Bosch J Burroughs AK. Liver cirrhosis. Lancet. (2014) 383:1749–61. 10.1016/S0140-6736(14)60121-5 24480518

[2] Albillos A Lario M Alvarez-Mon M. Cirrhosis-associated immune dysfunction: distinctive features and clinical relevance. J Hepatol. (2014) 61:1385–96. 10.1016/j.jhep.2014.08.010 25135860

[3] Acharya C Bajaj JS. Altered microbiome in patients with cirrhosis and complications. Clin Gastroenterol Hepatol. (2019) 17:307–21. 10.1016/j.cgh.2018.08.008 30099098 PMC6314917

[4] Irvine KM Ratnasekera I Powell EE Hume DA Causes and consequences of innate immune dysfunction in cirrhosis. Front Immunol. (2019) 10:293 10.3389/fimmu.2019.00818 30873165 PMC6401613

[5] Tritto G Bechlis Z Stadlbauer V Davies N Frances R Shah N. Evidence of neutrophil functional defect despite inflammation in stable cirrhosis. J Hepatol. (2011) 55:574–81. 10.1016/j.jhep.2010.11.034 21236309

[6] Zimmermann HW Seidler S Nattermann J Gassler N Hellerbrand C Zernecke A. Functional contribution of elevated circulating and hepatic non-classical CD14CD16 monocytes to inflammation and human liver fibrosis. PLoS ONE. (2010) 5:e11049. 10.1371/journal.pone.0011049 20548789 PMC2883575

[7] Doi H Iyer TK Carpenter E Li H Chang KM Vonderheide RH. Dysfunctional B-cell activation in cirrhosis resulting from hepatitis C infection associated with disappearance of CD27-positive B-cell population. Hepatology. (2012) 55:709–19. 10.1002/hep.24689 21932384 PMC3245804

[8] Chang LY Li Y Kaplan DE. Endotoxemia contributes to CD27+ memory B-cell apoptosis via enhanced sensitivity to Fas ligation in patients with Cirrhosis. Sci Rep. (2016) 6:36862. 10.1038/srep36862 27857173 PMC5114671

[9] Doi H Hayashi E Arai J Tojo M Morikawa K Eguchi J Enhanced B-cell differentiation driven by advanced cirrhosis resulting in hyperglobulinemia. J Gastroenterol Hepatol. (2018) 33:1667–76. 10.1111/jgh.14123 PMC6107433 29427373

[10] Lario M Munoz L Ubeda M Borrero MJ Martinez J Monserrat J. Defective thymopoiesis and poor peripheral homeostatic replenishment of T-helper cells cause T-cell lymphopenia in cirrhosis. J Hepatol. (2013) 59:723–30. 10.1016/j.jhep.2013.05.042 23742913

[11] Tian Z Chen Y Gao B. Natural killer cells in liver disease. Hepatology. (2013) 57:1654–62. 10.1002/hep.26115 23111952 PMC3573257

[12] Lewis SM Williams A Eisenbarth SC. Structure and function of the immune system in the spleen. Sci Immunol. (2019) 4:eaau6085. 10.1126/sciimmunol.aau6085 30824527 PMC6495537

[13] Lv Y Yee Lau W Wu H Han X Gong X Liu N. Causes of peripheral cytopenia in hepatitic cirrhosis and portal hypertensive splenomegaly. Exp Biol Med. (2017) 242:744–9. 10.1177/1535370217693113 28299974 PMC5363688

[14] Boyer TD Habib S Big spleens and hypersplenism: fix it or forget it? Liver Int. (2015) 35:1492–8. 10.1111/liv.12702 25312770

[15] Ueno H. Human circulating T follicular helper cell subsets in health and disease. J Clin Immunol. (2016) 36 (Suppl 1):34–9. 10.1007/s10875-016-0268-3 26984851

[16] Song W Craft J. T follicular helper cell heterogeneity: time, space, and function. Immunol Rev. (2019) 288:85–96. 10.1111/imr.12740 30874350 PMC6422039

[17] Kim ST Choi JY Lainez B Schulz VP Karas DE Baum ED. Human extrafollicular CD4(+) Th cells help memory B cells produce Igs. J Immunol. (2018) 201:1359–72. 10.4049/jimmunol.1701217 30030323 PMC6112860

[18] Greczmiel U Oxenius A. The janus face of follicular T helper cells in chronic viral infections. Front Immunol. (2018) 9:1162. 10.3389/fimmu.2018.01162 29887868 PMC5982684

[19] Lindqvist M van Lunzen J Soghoian DZ Kuhl BD Ranasinghe S Kranias G. Expansion of HIV-specific T follicular helper cells in chronic HIV infection. J Clin Invest. (2012) 122:3271–80. 10.1172/JCI64314 22922259 PMC3428098

[20] Lei Y Hu T Song X Nie H Chen M Chen W. Production of autoantibodies in chronic hepatitis B virus infection is associated with the augmented function of blood CXCR5^+^CD4^+^ T cells. PLoS ONE. (2016) 11:e0162241. 10.1371/journal.pone.0162241 27612199 PMC5017876

[21] Hunziker L Recher M Macpherson AJ Ciurea A Freigang S Hengartner H. Hypergammaglobulinemia and autoantibody induction mechanisms in viral infections. Nat Immunol. (2003) 4:343–9. 10.1038/ni911 12627229

[22] Shi J Zhao J Zhang X Cheng Y Hu J Li Y. Activated hepatic stellate cells impair NK cell anti-fibrosis capacity through a TGF-beta-dependent emperipolesis in HBV cirrhotic patients. Sci Rep. (2017) 7:44544. 10.1038/srep44544 28291251 PMC5349579

[23] Zhao J Zhang Z Luan Y Zou Z Sun Y Li Y. Pathological functions of interleukin-22 in chronic liver inflammation and fibrosis with hepatitis B virus infection by promoting T helper 17 cell recruitment. Hepatology. (2014) 59:1331–42. 10.1002/hep.26916 24677193 PMC3970185

[24] Wang L Sun Y Zhang Z Jia Y Zou Z Ding J. CXCR5^+^ CD4^+^ T follicular helper cells participate in the pathogenesis of primary biliary cirrhosis. Hepatology. (2015) 61:627–38. 10.1002/hep.27306 25042122 PMC4507804

[25] Havenar-Daughton C Lindqvist M Heit A Wu JE Reiss SM Kendric K. CXCL13 is a plasma biomarker of germinal center activity. Proc Natl Acad Sci USA. (2016) 113:2702–7. 10.1073/pnas.1520112113 26908875 PMC4790995

[26] Yu D Ye L. A Portrait of CXCR5(+) follicular cytotoxic CD8(+) T cells. Trends Immunol. (2018) 39:965–79. 10.1016/j.it.2018.10.002 30377045

[27] Papp M Norman GL Vitalis Z Tornai I Altorjay I Foldi I. Presence of anti-microbial antibodies in liver cirrhosis–a tell-tale sign of compromised immunity? PLoS ONE. (2010) 5:e12957. 10.1371/journal.pone.0012957 20886039 PMC2944893

[28] Ricard L Jachiet V Malard F Ye Y Stocker N Riviere S. Circulating follicular helper T cells are increased in systemic sclerosis and promote plasmablast differentiation through the IL-21 pathway which can be inhibited by ruxolitinib. Ann Rheum Dis. (2019) 78:539–50. 10.1136/annrheumdis-2018-214382 30760472

[29] Szabo K Papp G Szanto A Tarr T Zeher M. A comprehensive investigation on the distribution of circulating follicular T helper cells and B cell subsets in primary Sjogren's syndrome and systemic lupus erythematosus. Clin Exp Immunol. (2016) 183:76–89. 10.1111/cei.12703 26358223 PMC4687513

[30] Barbet G Sander LE Geswell M Leonardi I Cerutti A Iliev I. Sensing microbial viability through bacterial RNA augments T follicular helper cell and antibody responses. Immunity. (2018) 48:584–598.e5. 10.1016/j.immuni.2018.02.015 29548673 PMC5924674

[31] Ugolini M Gerhard J Burkert S Jensen KJ Georg P Ebner F. Recognition of microbial viability via TLR8 drives TFH cell differentiation and vaccine responses. Nat Immunol. (2018) 19:386–96. 10.1038/s41590-018-0068-4 29556002

[32] Blander JM Barbet G. Exploiting vita-PAMPs in vaccines. Curr Opin Pharmacol. (2018) 41:128–36. 10.1016/j.coph.2018.05.012 29890457 PMC6110613

[33] Harker JA Lewis GM Mack L Zuniga EI. Late interleukin-6 escalates T follicular helper cell responses and controls a chronic viral infection. Science. (2011) 334:825–9. 10.1126/science.1208421 21960530 PMC3388900

