# Supplementary material for: Hyperactive Follicular Helper T Cells Contribute to Dysregulated Humoral Immunity in Patients With Liver Cirrhosis
Source: Front Immunol. 2019 Aug 13;10:1915. doi: 10.3389/fimmu.2019.01915 (PMC6700335; doi:10.3389/fimmu.2019.01915)
Supplement: Supplementary file 1 [file Data_Sheet_1.docx]

Supplementary Material

**Supplemental Figure 1 Disruption of CD8^+^ Tc cell subset distribution in patients with liver cirrhosis.** (A) Representative dot plots show the gating strategy of CD8^+^ T cell subsets basing on CXCR5, CCR6 and CXCR3 expression in spleen and peripheral blood. Values in the quadrants represent the percentages of each subset. Among CD8^+^ T cells, CXCR5^+^ Tch cells, CCR6^+^CXCR3^-^ Tc17 cells, CCR6^+^CXCR3^+^ Tc1/Tc17cells, CCR6^–^CXCR3^+^ Tc1 cells, CCR6^–^CXCR3^–^ Tc2 cells were defined, respectively. (B) Summarized data show the percentages of CD8^+^ T cell subsets in the peripheral blood from HC (n = 42), HBV-LC (n = 24), and Non-HBV-LC groups (n = 23) and in the spleen from HC (n = 22), HBV-LC (n = 27), and Non-HBV-LC groups (n = 13). Each dot represents one subject, and line represents mean value. *P* values are shown.

**Supplemental Figure 2** Representative dot plots show the proportion of PD-1^high^ICOS^+^ cells among various Th (A) and Tc (B) subsets in peripheral blood and spleen from a LC patient. Values in the quadrants represent the percentages of splenic PD-1^high^ICOS^+^ cells.

**Supplemental Figure 3 Peripheral Tfh cells were significantly increased in LC patients.** (A) Representative dot plots show the proportion of PD-1^high^ICOS^+^ cells among CD185^+^CD4^+^ Tfh-like cells and CD185^+^CD8^+^ Tch cells in peripheral blood from a LC patient. Values in the gate represent the percentages of peripheral Tfh and Tch cell subset. (B) Summarized data show the percentages of PD-1^high^ICOS^+^CD185^+^CD4^+^ T cells and PD-1^high^ICOS^+^CD185^+^CD8^+^ T cells in the peripheral blood from HC (n = 42), HBV-LC (n = 24), and Non-HBV-LC groups (n = 23). Each dot represents one subject, and line represents mean value. ****P* < 0.001.

**Supplemental Figure 4 Tfh-like cell subsets were disturbed in both peripheral blood and spleen of patients with liver cirrhosis.** (A) Representative dot plots show the gating strategy of CXCR5^+^CD4^+^ T cell subsets basing on CCR6 and CXCR3 expression in spleen and peripheral blood. Values in the quadrants represent the percentages of each subset. Within CXCR5^+^CD4^+^ Tfh-like cells, CCR6^+^CXCR3^–^Tfh17 cells, CCR6^+^CXCR3^+^ Tfh1/Tfh17cells, CCR6^–^CXCR3^+^ Tfh1 cells, CCR6^–^CXCR3^–^ Tfh2 cells were defined, respectively. (B) Summarized data show the percentages of CXCR5^+^CD4^+^ Tfh-like cells in the peripheral blood from HC (n = 10), HBV-LC (n = 24), and Non-HBV-LC groups (n = 12) and in the spleen from HC (n = 22), HBV-LC (n = 27), and Non-HBV-LC groups (n = 13). Each dot represents one subject, and line represents mean value. *P* values are shown.
