# Supplementary figures and images for: Hyperactive Follicular Helper T Cells Contribute to Dysregulated Humoral Immunity in Patients With Liver Cirrhosis
Source: Front Immunol. 2019 Aug 13;10:1915. doi: 10.3389/fimmu.2019.01915 (PMC6700335; doi:10.3389/fimmu.2019.01915)

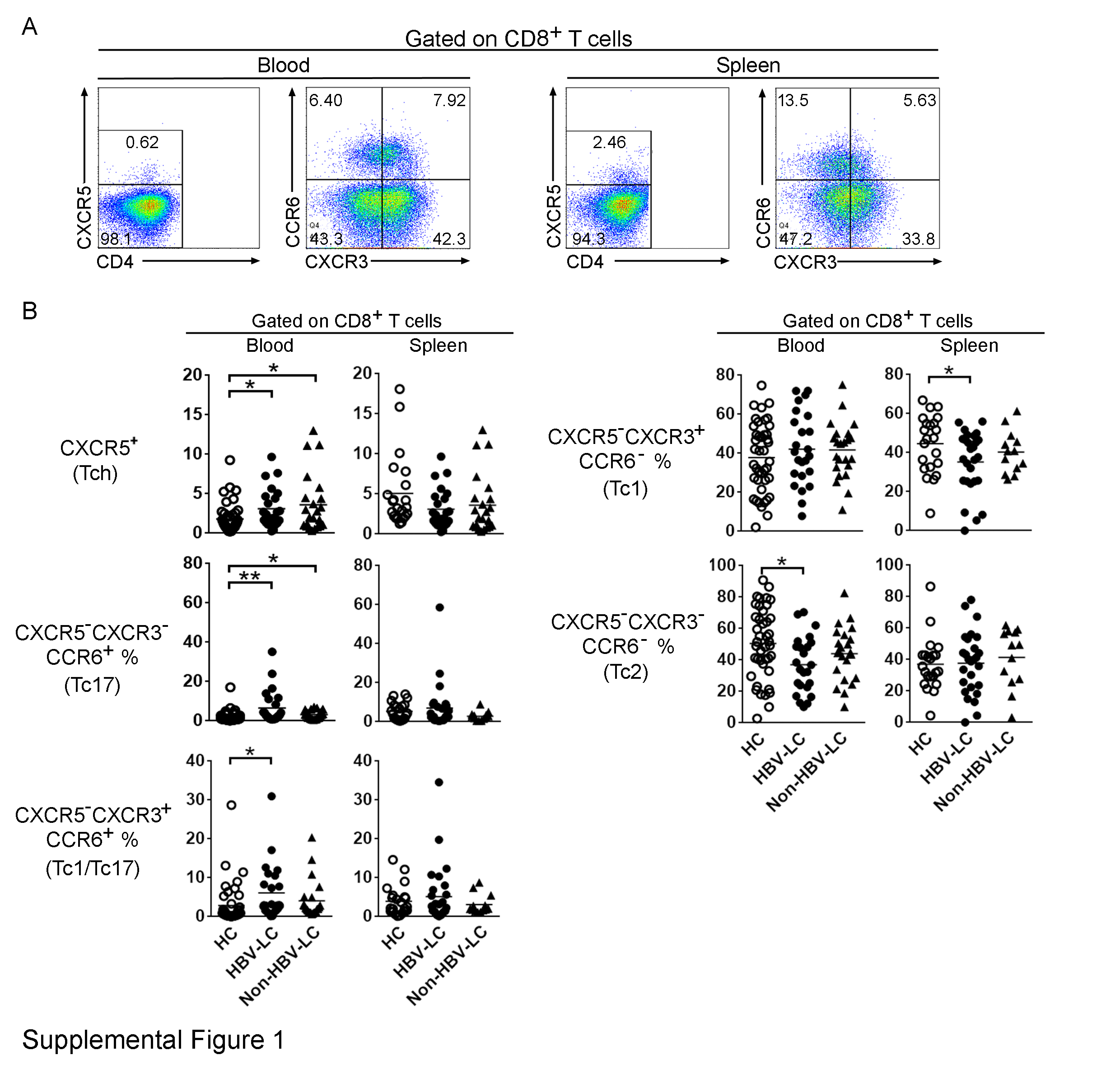

Supplement: Supplementary file 2 [file Image_1.TIF]

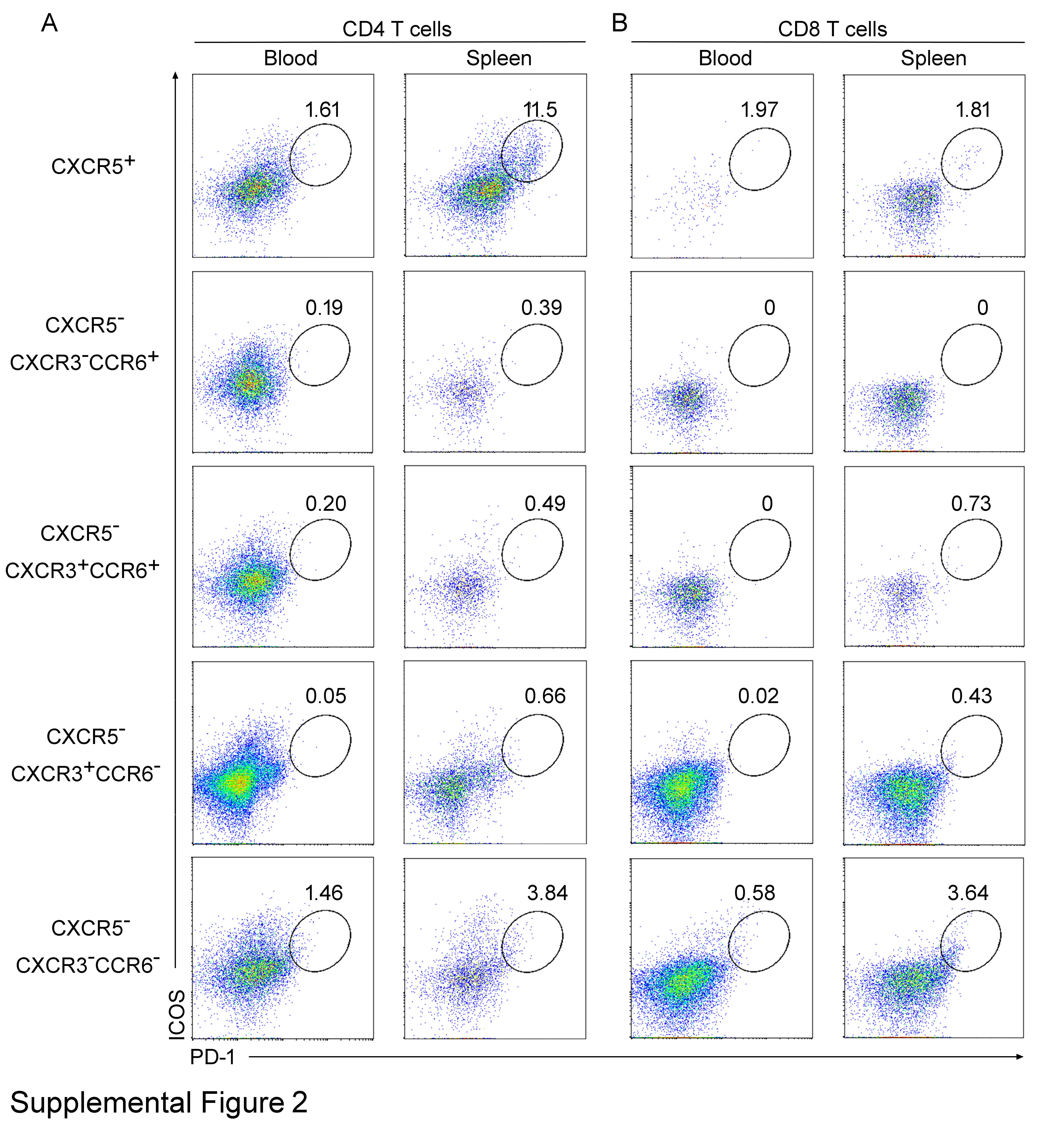

Supplement: Supplementary file 3 [file Image_2.TIF]

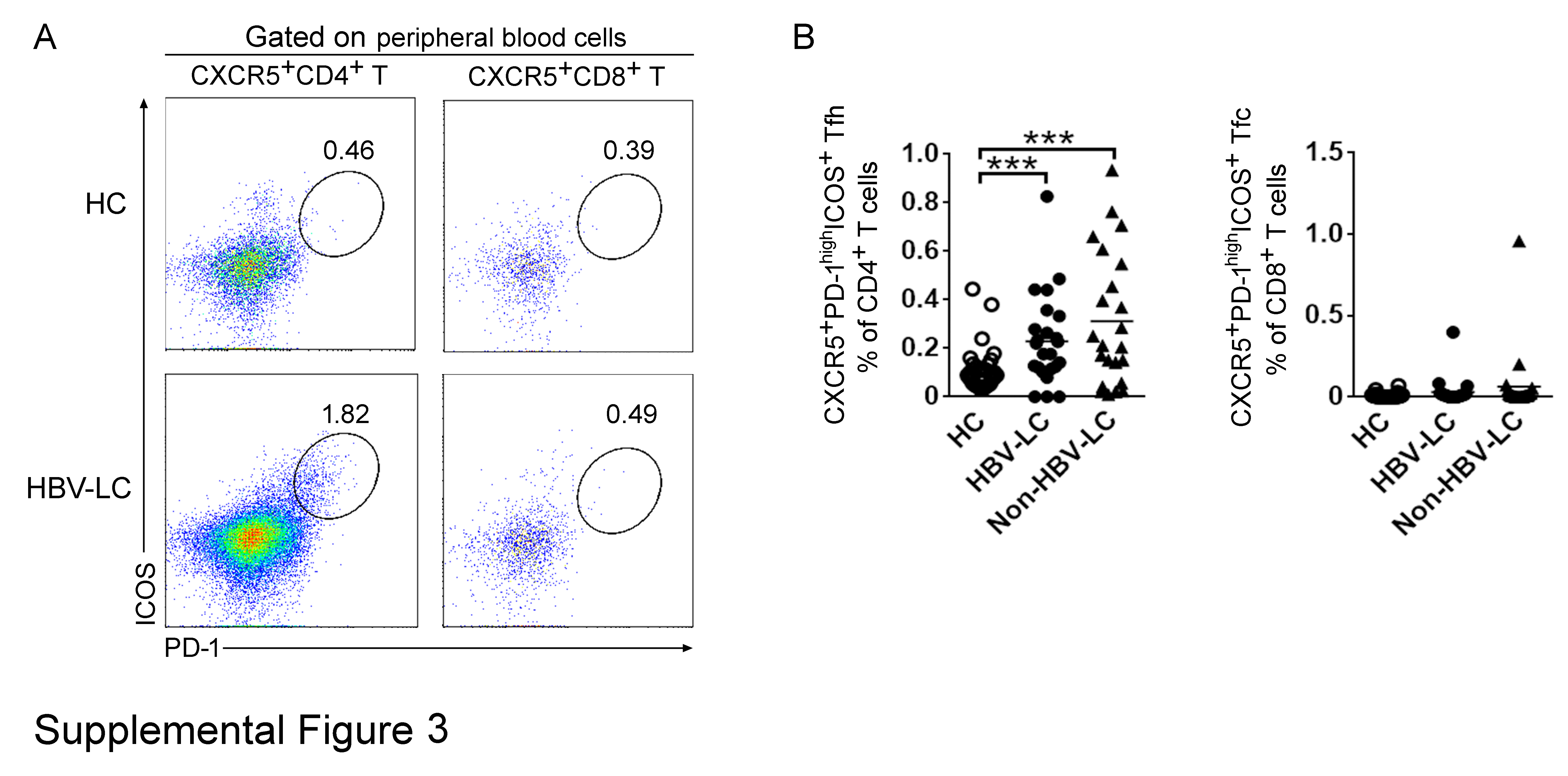

Supplement: Supplementary file 4 [file Image_3.TIF]

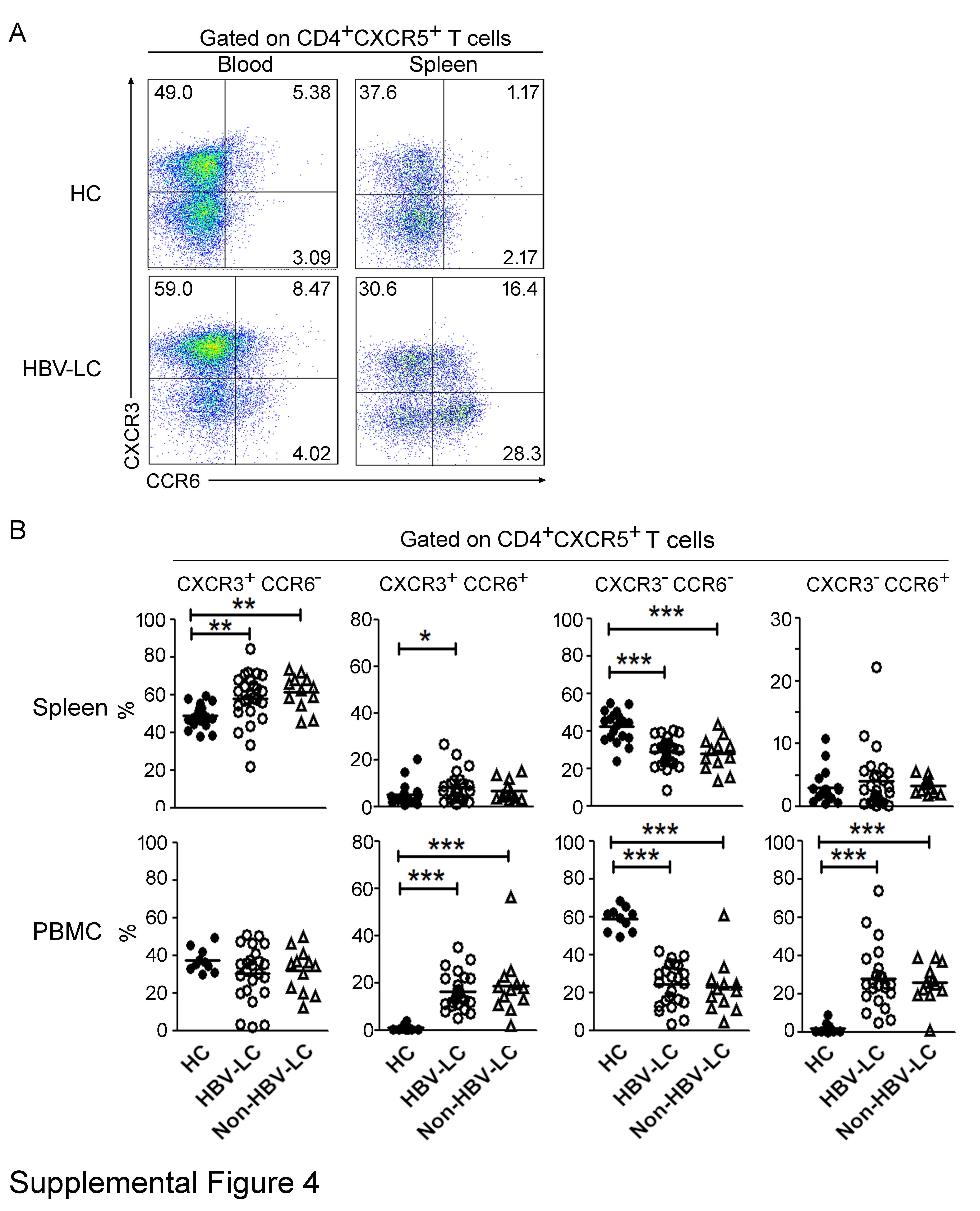

Supplement: Supplementary file 5 [file Image_4.TIF]
